# In Vitro Antimicrobial Effectiveness Tests Using Garlic (*Allium sativum*) against *Salmonella enterica* Subspecies *enterica* Serovar Enteritidis

**DOI:** 10.3390/antibiotics11111481

**Published:** 2022-10-26

**Authors:** Elena Circella, Gaia Casalino, Francesco D’Amico, Nicola Pugliese, Michela Maria Dimuccio, Antonio Camarda, Giancarlo Bozzo

**Affiliations:** Department of Veterinary Medicine, University of Bari “Aldo Moro”, S.P. per Casamassima, km 3, 70010 Valenzano, Italy

**Keywords:** *Salmonella enteritidis*, laying hens, garlic, antimicrobial properties

## Abstract

In recent years, there has been a more prudent use of drugs on livestock farms and alternative products have been considered, with a view of reducing the risk of the onset of antibiotic resistance. *Salmonella enterica* subsp. *enterica* serovar Enteritidis (*S.* Enteritidis) may cause disease in poultry, and it is also responsible for human food poisoning. The aim of this study was to evaluate the efficacy of garlic against *S.* Enteritidis and to define its Minimal Inhibitory Concentration (MIC)_90_ and MIC_50_ values. The study was carried out in vitro, testing 26 *S.* Enteritidis strains identified in laying hens from various farms in Southern Italy. A preliminary efficacy trial was carried out on two strains, *S.* Enteritidis and *Escherichia coli*, using a garlic concentration of 10 mg/mL (1%). Later, 26 strains of *S.* Enteritidis at 10^6^ Colony Forming Unit (CFU) and 10^4^ CFU were tested with different concentrations of garlic, ranging from 10 mg/mL to 1 mg/mL. Based on the results, intermediate concentrations of garlic, from 5 mg/mL to 4 mg/mL and 4 mg/mL to 3 mg/mL, were used to test 10^6^ CFU and 10^4^ CFU, respectively. The data were statistically analyzed. The MIC_90_ was 4.75 mg/mL for strains tested at 10^6^ CFU and 4 mg/mL for strains tested at 10^4^ CFU. The results highlight garlic’s potential to inhibit the growth of *Salmonella enterica* ser. Enteritidis in vitro. Efficacy was dependent on the microbial concentration used. In vivo efficacy trials will be crucial to confirm the efficacy of garlic against *S.* Enteritidis and to assess whether garlic can be used in poultry flocks to prevent the spread of the bacterium in the field.

## 1. Introduction

In recent years, due to fears surrounding the risk of antibiotic resistance, there has been growing interest in alternative methods to pharmaceutical approaches for controlling potential pathogens on livestock farms. Antimicrobials are used in herds both as a treatment and for prophylactic purposes. The misuse of drugs has contributed to the onset of antimicrobial resistance (AMR), especially in the past. AMR is the consequence of the evolutionary response and evasive strategies that bacteria exhibit in response to a selection pressure caused by an external insult, such as exposure to antibiotics. This leads to the expression of mutations or the transmission of mobile genetic elements through horizontal gene transfer (HGT) among bacterial populations [1]. Currently, the greatest risk is the spread of so-called ‘superbugs’, i.e., multidrug-resistant bacteria (MDR).

On the basis of Regulation (EU) 2016/429 of the European Parliament and of the Council of 9 March 2016 on transmissible animal diseases and amending and repealing certain acts in the area of animal health (Animal Health Law) [2], biosecurity measures have been increased, health and hygiene standards within farms have been raised, animal densities have been regulated, and there has been a greater use of vaccine prophylaxis even for the prevention of some bacterial forms. 

The possibility of using natural substances, or their derivatives, as an alternative to the use of antibiotics has also prompted the scientific community to investigate their potential effectiveness.

Ginger (*Zingiber officinale*) and turmeric (*Curcuma longa*) have antibacterial properties [3]. Cinnamon (*Cinnamomum verum*) has been used as a natural treatment for nausea, colds and enteric manifestations attributed to viruses [4]. Aloe (*Aloe vera*) appears to have anti-inflammatory, antioxidant, hypoglycemic and antibacterial properties [5]. Thanks to its components, neem (*Azadirachta indica*) has antifungal, antipyretic, anti-inflammatory, antiarthritic, diuretic and immunomodulatory effects [6] and has also shown effects against parasites, insects and bloodsucking arthropods [7]. Even the essential oil of mint (*Mentha arvensis*) has effective antibacterial properties, in particular against *Salmonella* enterica subsp. *enterica* serovar Enteritidis and *Listeria monocytogenes* [8].

Garlic (*Allium sativum*), belonging to the *Liliaceae* family, is an aromatic herbaceous annual crop and one of the most important herbs in traditional medicine [9,10]. *Allium* species and their components have been described as having antioxidant [11], antitumoral [12], anti-inflammatory [13], immunomodulatory [14], antiviral [15], antimicrobial [16] and cardiovascular defensive actions [17]. Moreover, it possesses therapeutic purposes, including the treatment of lung disorders, whooping cough, stomach disorders, colds and earache [18].

The health benefits associated with the use of garlic are attributed to its anticancer, anti-inflammatory, antifungal, antiviral and antibacterial properties. Several in vitro, in vivo and epidemiological studies indicate that garlic exhibits anticancer activity, and its likely mechanism of action is the activation of metabolizing enzymes, inhibiting reactive oxygen species and radical scavenging, preventing DNA damage and inhibiting tumors [19]. Its immunomodulatory effects are mediated through its ability to modulate cytokine production as well as to activate immune responses by stimulating antibody secretion and immune cells [20].

Garlic’s antimicrobial, antiviral and fungitoxic properties against different pathogens have been shown [21,22]. 

Garlic contains different bioactive complexes, and organosulfur compounds are its main bioactive components, with allicin, which is responsible for garlic’s strong odor, being the most commonly described substance with medicinal activity in the literature [23,24]. Allicin was recognized by Cavallito and Bailey [25] and has been shown to have antimicrobial activities against Gram-positive and Gram-negative bacteria [26]. It is transformed from its precursor alliin by the enzyme alliinase. Being rather unstable, it soon participates in a cascade of non-enzymatic reactions, producing such compounds as vinyl dithiins, ajoene and polysulphides, which have been reported to exhibit antimicrobial activity [23,27].

Its antibacterial activity has also been well-recognized in different foods [28]. Eating garlic reduced *S. enterica* populations in mayonnaise by 10-fold [29]. Furthermore, garlic integrated in butter improved the inactivation of Salmonella (5 log CFU/g), *E. coli* O157:H7 (1.5 log CFU/g) and *L. monocytogenes* (2 log CFU/g) after 48 h of storage at 21 °C [30].

Taking into account such potential, the aim of our study was to evaluate how effective garlic might be against strains of *Salmonella enterica* ser. Enteritidis. This bacterium is a pathogen for poultry, in which it can induce morbid conditions characterized by enteritis, production losses and mortality while, via contamination of meat and eggs, it is also responsible for food poisoning in humans [31].

## 2. Material and Methods

### 2.1. Strains Used for Bacterial Suspensions 

The study was carried out in vitro on 26 *Salmonella* enterica subsp. *enterica* ser. Enteritidis previously isolated in laying hen farms for the production of eggs for consumption, following the methodology proposed by Mooijman et al. [32]. These strains were stored at −20 °C in Brucella broth and glycerol (10%) at the Department of the Veterinary Medicine, University of Bari (Valenzano, Italy).

All strains were grown on Tryptic Soy Agar (TSA) (OXOID, Basingstoke, UK) at 37 °C overnight before testing. Starting from each strain, two bacterial suspensions were prepared with respective concentrations of 1 × 10^6^ CFU/mL and 1 × 10^4^ CFU/mL. To obtain the predefined concentrations, according to CLSI standards [33], bacterial suspensions equal to the 0.5 McFarland standard were prepared, corresponding to 1–2 × 10^8^ CFU/mL. The concentrations obtained were confirmed by serial dilutions and plate bacterial counts. Ten microliters of the suspensions with a concentration of 10^8^ CFU/mL was inoculated in spots, in order to analyze in the tests a charge equal to 10^6^ CFU. Meanwhile, for the tests using 10^4^ CFU, suspensions at 10^8^ CFU/mL were diluted stepwise in sterile 0.9% saline solution to obtain 10^6^ CFU/mL, and 10 µL was then inoculated in each spot.

### 2.2. Preparation of Aqueous Garlic Extract (AGE)

For the efficacy trials, commercial freeze-dried garlic was used. The lyophilized powder was weighed and placed in 100 mL of sterile distilled water in different quantities (10 g, 9 g, 8 g, etc.) based on the different concentrations of garlic that we intended to test. The efficacy tests were performed on Mueller–Hinton agar (OXOID), reconstituted using 900 mL of water, sterilized in an autoclave at 121 °C and subsequently brought to 50 °C before adding garlic suspensions at the different concentrations to the medium.

### 2.3. Preliminary Test

A 1% solution, with a garlic concentration of 10 mg/mL, was used for a first preliminary efficacy test on a strain of *Escherichia coli* (ATCC 25922) and a strain of *Salmonella enterica* subsp. *enterica* ser. Enteritidis. From the bacteria, 10^8^ CFU/mL suspensions were prepared and inoculated in a plate containing simple Mueller–Hinton agar to which garlic was added, using common inoculation methods: with a bacteriological loop, with a sterile swab and by spot.

**Figure 1:**
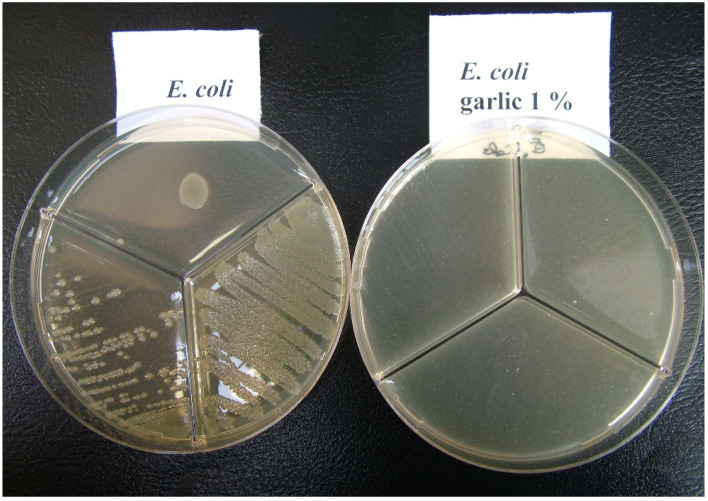
*Escherichia coli (E. coli*). **Left** side: bacterial growth on the medium without garlic. **Right** side: total inhibition of growth in presence of 1% garlic.

**Figure 2:**
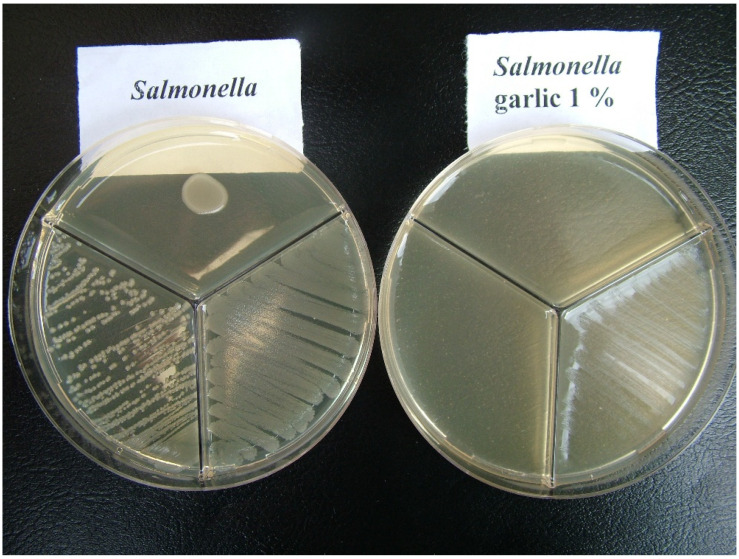
*Salmonella*. **Left** side: bacterial growth on the medium without garlic. **Right** side: total inhibition of growth in presence of 1% garlic.

The presence of garlic in the added medium resulted in total inhibition of growth of both *E. coli* and *Salmonella*, regardless of the method of inoculation (Figure 1 and Figure 2).

### 2.4. Preparation of Efficacy Tests

**Figure 3:**
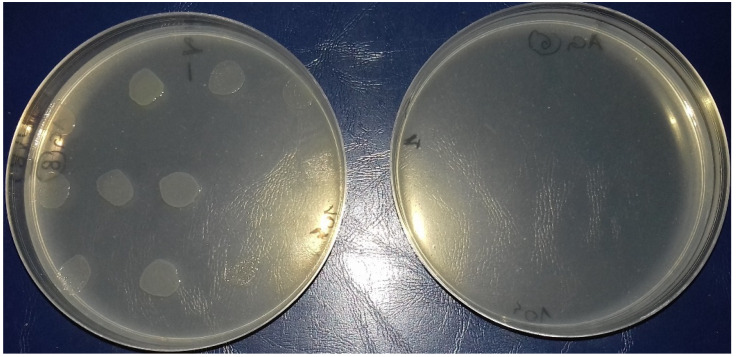
*Salmonella* strains. **Left** side: growth/no growth of the strains. **Right** side: total inhibition of growth in presence of 1% garlic used as inhibition control.

**Figure 4:**
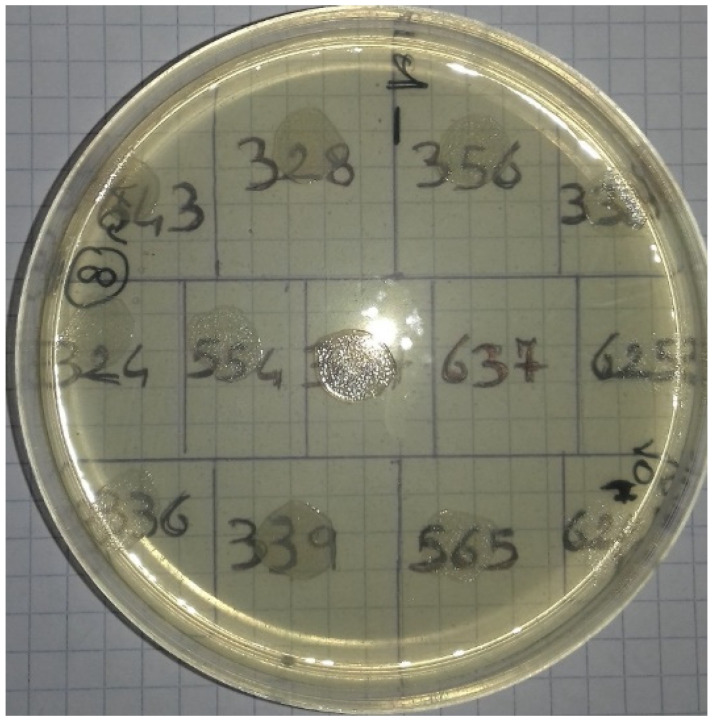
*Salmonella* strains. Numbered grid used for the identification of strains.

Several series of Mueller–Hinton agar plates were prepared, containing concentrations of garlic from 10 mg/mL to 1 mg/mL (10 mg/mL; 9 mg/mL; 8 mg/mL; 7 mg/mL; 6 mg/mL; 5 mg/mL; 4 mg/mL; 3 mg/mL; 2 mg/mL; 1 mg/mL), which were used to test both bacterial concentrations (10^6^ CFU and 10^4^ CFU) for each strain. The plates were incubated at 37 °C, and the results were read after 24 h of incubation, evaluating the efficacy of AGE based on growth/no growth in the *spot* (Figure 3). Each strain was identified using a numbered grid placed under the plate (Figure 4).

Each experiment was carried out twice on two different days. Based on the results obtained, the bacterial loads at 10^6^ CFU were evaluated with a range of garlic concentrations from 5 mg/mL to 3.5 mg/mL (5 mg/mL; 4.75 mg/mL; 4.5 mg/mL; 4.25 mg/mL; 4 mg/mL; 3.75 mg; 3.5 mg) and at 10^4^ CFU with concentrations from 4 mg/mL to 3.25 mg/mL (4 mg/mL; 3.75 mg/mL; 3.25 mg/mL).

### 2.5. Statistical Data Evaluation

The data obtained from the different efficacy tests were compared to evaluate their statistical significance using the two-sided Fisher test. Statistical analysis was carried out in R v. 4.2.1 [34].

## 3. Results 

Garlic concentrations greater than or equal to 5 mg/mL were found to be effective, inhibiting the growth of all strains regardless of the bacterial concentration tested. MIC_90_ and MIC_50_ values were found to be 5 mg/mL at 10^6^ CFU, while only 6 of 26 strains (23.07%) were completely inhibited by a concentration of 4 mg/mL (Table 1). At 10^4^ CFU, the corresponding MIC_90_ and MIC_50_ values were 4 mg/mL, with the growth of 24 out of 26 strains (92.3%) being inhibited. 

A concentration of 3 mg/mL proved to be ineffective for all the strains analyzed at a concentration of 10^6^ CFU, while at 10^4^ CFU, the growth of just one bacterial strain was inhibited.

Analyzing the effectiveness of the intermediate concentrations of garlic, from 5 mg/mL to 3.5 mg/mL for the 10^6^ CFU inoculations (Table 2), the minimum concentration inhibiting the growth of the strains in 90% of cases (MIC_90_) was found to be 4.75 mg/mL, while a concentration of 4.5 mg/mL was not as effective (*p* < 0.001), as less than 50% of the strains were inhibited at this concentration.

At 10^4^ CFU, MIC_90_ stood at 4 mg/mL, while the concentration of 3.75 mg/mL inhibited the growth of 22 out of 26 strains, i.e., well above 50% (MIC_50_) and significantly higher than at 3.5 mg/mL (*p* = 0.034). Comparing the two different bacterial concentrations used, an inhibitory effect at 4 mg/mL was observed in one strain equal to 10^4^ CFU. However, the same concentration did not have the same efficacy on suspensions at 10^6^ CFU (*p* < 0.001).

## 4. Discussion

The results show that garlic possesses a good inhibitory capacity in vitro against *Salmonella* enterica sub. *enterica* ser. Enteritidis. This capacity was variable and dependent on the bacterial load used. Given that the rate of bacteria inhibition is not necessarily proportional to the concentration of AGE in a linear way, the same decrease in the rates is not expected to be observed when concentrations are decreased from 5 to 4.75 mg/mL vs. when they are decreased from 4.75 mg/mL to 4.5 mg/mL, etc., with 0.25 mg/mL intervals.

The inhibitory capacity of garlic is predominantly due to its bacteriostatic rather than bactericidal properties, as MIC values are generally below the Minimum Bactericidal Concentration (MBC) [35].

Studies on the antimicrobial efficacy of garlic, conducted on individual strains by bacterial species, have shown in vitro MIC values of 6.25 mg/mL for *Streptococcus mutans* [36] and for *Salmonella* Tiphi, *Escherichia coli*, *Staphylococcus aureus* and *Pseudomonas* [37]. These are all higher than the MIC_90_ values obtained in this study for *S.* e. sub *e.* ser. Enteritidis. 

In another experiment [38], also including only one strain of *Salmonella* Tiphi, the MIC value obtained was much lower, at 0.02 mg/mL. Such divergences could be attributable to the individual sensitivity of a particular strain, since in these studies there was no comparison with other strains and the bacterial load adopted.

When evaluating the effectiveness of natural substances, unlike in the pharmacological field, any divergence among the values obtained may be linked to a lack of standardization of the methods adopted in the different laboratories. For example, in the two studies cited on *Salmonella* Tiphi, in one case [37], the evaluation was carried out through serial dilutions in broth, while in the other case [38], the MIC was determined in a plate in solid medium according to the Kirby–Bauer method, soaking discs with garlic extracts specially constructed for the experiment. Furthermore, the different concentration panels adopted in the two studies led—in the research conducted by Andualem [37]—to MIC values of 6.25 mg/mL, corresponding to the lowest concentration of garlic evaluated in the trial, while, on the other hand, Gull et al. [38], who evaluated even lower concentrations, obtained MIC concentrations ranging from 0.02 to 0.2 mg/mL for the bacteria tested. 

In a larger study, involving 25 strains of *E. coli* and *S. aureus* analyzed in bacterial concentrations of 10^7^ CFU and by serial dilutions in broth, the MIC values were between 4 mg/mL and 8 mg/mL for both bacteria [39].

Other factors that can influence the discrepancies found in different studies may include the instability of allicin; the active ingredient responsible for its antimicrobial activity against Gram-positive and Gram-negative bacteria; or else the physical form of garlic used, for example, fresh or dried, and, when using an extract, the type and method of preparation [26,40]. Allicin is transformed from its precursor alliin by the enzyme alliinase. Being rather unstable, it soon participates in a cascade of non-enzymatic reactions, producing such compounds as vinyl dithiins, ajoene and polysulphides, which have been reported to exhibit antimicrobial activity [23,27].

Comparative tests with garlic extracts, i.e., aqueous, alcoholic in ethanol and alcoholic in methanol, showed better efficacy of the aqueous extract compared to the alcoholic ones, whose efficacy can be superimposed [38], but these results were variable depending on the germ tested [35]. AGE exhibited in vitro antibacterial activity against various pathogenic bacteria including *Bacillus cereus*, *Shigella* and *Salmonella* species and enterotoxigenic *E. coli* [41].

The antimicrobial efficacy of natural substances could be enhanced by their combination [26,42]. For example, thyme, peppermint, sage, black pepper and garlic appear to have a greater in vitro antimicrobial effect against *Bacillus subtilis* and *Salmonella* Enteritidis if tested in synergy rather than individually [26,43], although Andualem [37] showed no real enhancement of garlic’s efficacy when it was combined with honey.

Field tests carried out on groups of turkeys have shown the effectiveness of a mixture composed of organic acids (acetic, formic and propionic acid), and cinnamic aldehyde, a cinnamon extract, added to the feed at a concentration of 0.2% in reducing the intestinal concentration of *E. coli* and in limiting the lesions induced by a 078 antibiotic-resistant strain [44].

## 5. Conclusions

The results obtained in this preliminary study are quite encouraging. However, further evaluations on a larger number of strains and on possible combinations of garlic with other natural substances are desirable in order to assess its applicability. Indeed, its combination with other natural substances could help to limit the amount of garlic administered to animals, thus reducing the risk of transmitting unpleasant odors to feed and animal-derived products, such as eggs or meat.

## Figures and Tables

**Table 1:** In vitro efficacy of AGE with concentrations ranging from 10 mg/mL to 1 mg/mL).

Extract Concentration (mg/mL)	N° Inhibited Strains/Analyzed Strains (%)	
	10^6^ CFU *	10^4^ CFU *
10	26/26 (100)	26/26 (100)
9	26/26 (100)	26/26 (100)
8	26/26 (100)	26/26 (100)
7	26/26 (100)	26/26 (100)
6	26/26 (100)	26/26 (100)
5	26/26 (100)	26/26 (100)
4	6/26 (23.07)	24/26 (92.3)
3	0/26 (0)	1/26 (3.84)
2	0/26 (0)	0/26 (0)
1	0/26 (0)	0/26 (0)

* Bacterial load used.

**Table 2:** In vitro efficacy of AGE with concentrations ranging from 5 mg/mL to 3.5 mg/mL and from 4 mg/mL and 3.25 mg/mL.

Extract Concentration (mg/mL)	N° Inhibited Strains/Analyzed Strains (%)	
	10^6^ CFU *	10^4^ CFU *
5	26/26 (100)	-
4.75	25/26 (96.15)	-
4.5	9/26 (34.61)	-
4.25	6/26 (23.07)	-
4	6/26 (23.07)	24/26 (92.3)
3.75	0/26 (0)	22/26 (84.61)
3.5	0/26 (0)	11/26 (42.3)
3.25	-	1/26 (3.84)

* Bacterial load used.

## Data Availability

Data is contained within the article.

## References

[1] Reygaert W.C. (2018). An overview of the antimicrobial resistance mechanisms of bacteria. AIMS Microbiol..

[2] Regulation (EU) No 2016/429 of the European Parliament and of the Council of 9 March 2016 on Transmissible Animal Diseases and Amending and Repealing Certain Acts in the Area of Animal health (Animal Health Law). https://www.legislation.gov.uk/eur/2016/429/introduction.

[3] Ibrahim S.A., Dharmavavaram S.R., Seo C.W., Shahbazi G. (2004). Antimicrobial activity of Bididobacterium Longum (NCFB2259) as influenced by spices. Internet. J. Food. Saf..

[4] Sharifi-Rad J., Dey A., Koirala N., Shaheen S., Omari N.E., Salehi B., Goloshvili T., Silva N.C.C., Bouyahya A., Vitalini S. (2021). Cinnamomum Species: Bridging Phytochemistry Knowledge, Pharmacological Properties and Toxicological Safety for Health Benefits. Front. Pharmacol..

[5] Fani M.M., Kohanteb J. (2012). Inhibitory activity of Aloe vera gel on some clinically isolated cariogenic and periodontopathic bacteria. J. Oral. Sci..

[6] Biswas K., Chattopadhyay I., Banerjee R.K., Bandyopadhyay U. (2002). Biological activities and medicinal properties of neem (Azadirachta indica). India Curr. Sci..

[7] Camarda A., Pugliese N., Bevilacqua A., Circella E., Gradoni L., George D., Sparagano O., Giangaspero A. (2018). Efficacy of a novel neem oil formulation (RP03™) to control the poultry red mite Dermanyssus gallinae. Med. Vet. Entomol..

[8] Tassou C.C., Drosinos E.H., Nychas G.J.E. (1995). Effects of essential oil from mint (Mentha piperita) on Salmonella enteritidis and Listeria monocytogenes in model food system at 4 degrees and 10 degrees C. J. Appl. Bacteriol..

[9] Ayaz E., Alposy H.C. (2007). Garlic (*Allium sativum*) and traditional medicine. Turk. Parazitol. Derg..

[10] Badal D.S., Dwivedi A.K., Kumar V., Singh S., Prakash A., Verma S., Kumar J. (2019). Effect of organic manures and inorganic fertilizers on growth, yield and its attributing traits in garlic (*Allium sativum* L.). J. Pharmacogn. Phytochem..

[11] Capasso A. (2013). Antioxidant action and therapeutic efficacy of *Allium sativum* L.. Molecules.

[12] Nicastro H.L., Ross S.A., Milner J.A. (2015). Garlic and onions: Their cancer prevention properties. Cancer Prev. Res..

[13] Lee D.Y., Li H., Lim H.J., Lee H.J., Jeon R., Ryu J.H. (2012). Anti-inflammatory activity of sulfur-containing compounds from garlic. J. Med. Food.

[14] Percival S.S. (2016). Aged garlic extract modifies human immunity. J. Nutr..

[15] Mehrbod P., Amini E., Kheir M.T., Pasteur Institute of IRAN (Influenza Unit) (2009). Antiviral activity of garlic extract on influenza virus. Iran. J. Virol..

[16] Serrano H.D.A., Mariezcurrena-Berasain M.A., del Castillo A.C.G., Carranza B.V., Pliego A.B., Rojas M.T., Anele U.Y., Salem A.Z.M., Rivas-Caceres R.R. (2020). Antimicrobial resistance of three common molecularly identified pathogenic bacteria to Allium aqueous extracts. Microb. Pathog..

[17] Castro C., Lorenzo A.G., González A., Cruzado M. (2010). Garlic components inhibit angiotensin II-induced cell-cycle progression and migration: Involvement of cell-cycle inhibitor p27 (Kip1) and mitogen-activated protein kinase. Mol. Nutr. Food Res..

[18] Batiha G.E.-S., Beshbishy A.G.M., Wasef L., Elewa Y.H.A., Al-Sagan A.A., El-Hack M.E.A., Taha A.E., Abd-Elhakim Y.M., Devkota H.P. (2020). Chemical Constituents and Pharmacological Activities of Garlic (*Allium sativum* L.): A Review. Nutrients.

[19] Zhang C., Xie J., Li X., Luo J., Huang X., Liu L., Peng X. (2019). Alliin alters gut microbiota and gene expression of colonic epithelial tissues. J. Food Biochem..

[20] Arreola R., Quintero-Fabian S., Lopez-Roa R.I., Flores-Gutierrez E.O., ReyesGrajeda J.P., Carrera-Quintanar L., Ortuño-Sahagún D. (2015). Immunomodulation and anti-inflammatory effects of garlic compounds. J. Immunol. Res..

[21] Mylona K., Garcia-Cela E., Sulyok M., Medina A., Magan N. (2019). Influence of two garlic-derived compounds, propyl propane thiosulfonate (PTS) and propyl propane thiosulfinate (PTSO), on growth and mycotoxin production by Fusarium species in vitro and in stored cereals. Toxins.

[22] Khubber S., Hashemifesharaki R., Mohammadi M., Gharibzahedi S.M.T. (2020). Garlic (*Allium sativum* L.): A potential unique therapeutic food rich in organosulfur and flavonoid compounds to fight with COVID-19. Nutr. J..

[23] Quesada I., de Paola M., Torres-Palazzolo C., Camargo A., Ferder L., Manucha W., Castro C. (2020). Effect of garlic’s active constituents in inflammation, obesity and cardiovascular disease. Curr. Hypertens. Rep..

[24] Shang A., Cao S.-Y., Xu X.-Y., Gan R.-Y., Tang G.-Y., Corke H., Mavumengwana V., Li H.-B. (2019). Bioactive compounds and biological functions of garlic (*Allium sativum* L.). Foods.

[25] Cavallito C.J., Bailey J.H. (1944). Allicin, the antibacterial principle of *Allium sativum*. I. Isolation, physical properties and antibacterial action. J. Am. Chem. Soc..

[26] Choo S., Chin V.K., Wong E.H., Madhavan P., Tay S.T., Yong P.V.C., Chong P.P. (2020). Review: Antimicrobial properties of allicin used alone or in combination with other medications. Folia Microbiol..

[27] Nakamoto M., Kunimura K., Suzuki J.I., Kodera Y. (2020). Antimicrobial properties of hydrophobic compounds in garlic: Allicin, vinyldithiin, ajoene and diallyl polysulfides. Exp. Ther. Med..

[28] Wilson E., Demmig-Adams B. (2007). Antioxidant, anti-inflammatory, and antimicrobial properties of garlic and onions. Nutr. Food Sci..

[29] Leuschner R., Zamparini J. (2002). Effects of spices on growth and survival of Escherichia coli O157 and Salmonella enterica serovar Enteritidis in broth model systems and mayonnaise. Food Control.

[30] Adler B.B., Beuchat L.R. (2002). Death of Salmonella, Escherichia coli O157, H7, and Listeria monocytogenesin garlic butter as affected by storage temperature. J. Food Prot..

[31] Gast R.K., Jones D.R., Guraya R., Garcia J.S., Karcher D.M. (2022). Research Note: Internal organ colonization by Salmonella Enteritidis in experimentally infected layer pullets reared at different stocking densities in indoor cage-free housing. Poult. Sci..

[32] Mooijman K.A., Pielaat A., Kuijpers A.F. (2018). Validation of EN ISO 6579-1—Microbiology of the food chain—Horizontal method for the detection, enumeration and serotyping of Salmonella—Part 1 detection of Salmonella spp.. Int. J. Food Microbiol..

[33] CLSI (2006). Methods for Dilution Antimicrobial Susceptibility Tests for Bacteria that Grow Aerobically: Approved Standard.

[34] R Core Team (2022). R: A Language and Environment for Statistical Computing.

[35] Mohsenipour Z., Hassanshahian M. (2015). The effects of Allium sativum Extracts on Biofilm Formation and Activities of Six Pathogenic Bacteria. Jundishapur J. Microbiol..

[36] Jain I., Jain P., Bisht D., Sharma A., Srivastava B., Gupta N. (2015). Comparative Evaluation of Antibacterial Efficacy of Six Indian Plant Extracts against Streptococcus Mutans. J. Clin. Diagn. Res..

[37] Andualem B. (2013). Combined antibacterial activity of stingless bee (Apis mellipodae) honey and garlic (*Allium sativum*) extracts against standard and clinical pathogenic bacteria. Asian Pac. J. Trop. Biomed..

[38] Gull I., Saeed M., Shaukat H., Aslam S., Samra Z., Athar A. (2012). Inhibitory effect of allium satibum and Zingiber officinale extracts on clinically Important drug resistant pathogenic bacteria. Ann. Clin. Microbiol. Antimicrob..

[39] Yadav S., Trivedi N.A., Bhatt J.D. (2015). Antimicrobial activity of fresh garlic juice: An in vitro study. Pharmacol. Stud..

[40] Belguith H., Kthiri F., Chati A., Sofah A.A., Hamida J.B., Landoulsi A. (2010). Study of the effect of aqueous garlic extract (*Allium sativum*) one some Salmonella serovars isolates. Emir. J. Food Agric..

[41] Bhatwalkar S.B., Mondal R., Krishna S.B.N., Adam J.K., Govender P., Anupam R. (2021). Antibacterial Properties of Organosulfur Compounds of Garlic (*Allium sativum*). Front. Microbiol..

[42] Bag A., Chattopadhyay R.R. (2015). Evaluation of Synergistic Antibacterial and Antioxidant Efficacy of Essential Oils of Spices and Herbs in Combination. PLoS ONE.

[43] Al-Turki A.I. (2007). Antibacterial effect of thyme, peppermint, sage, black pepper, and garlic hydrosols against Bacillus subtilis and Salmonella enteritidis. J. Food Agric. Environ..

[44] Parigi M., Massi P., Fiorentini L., Tosi G., Romboli C., Vandi L., Bocciero R., Fregnani G. Valutazione dell’efficacia di una miscela di acidi organici e fitoterapici nel controllo dell’infezione da Escherichia coli nel tacchino. Proceedings of the Atti II Simposio Scientfico SIPA.

